# Vocal risk mapping in classical singers: an analysis of the self-perception of voice quality, vocal fatigue, and singing voice handicap

**DOI:** 10.1590/2317-1782/20242023088en

**Published:** 2024-06-14

**Authors:** Diógenes Gomes, Felipe Moreti, Mara Behlau

**Affiliations:** 1 Centro de Estudos da Voz - CEV - São Paulo (SP), Brazil.; 2 Speech-Language Pathology and Audiology Department, Faculdade de Filosofia e Ciências, Universidade Estadual Paulista "Júlio de Mesquita Filho" - Unesp - Marília (SP), Brazil.

**Keywords:** Voice, Singing, Self-Testing, Surveys and Questionnaires, Fatigue, Speech, Language and Hearing Sciences

## Abstract

**Purpose:**

To map the vocal risk in professional classical singers, analyzing their self-assessment of voice and self-perception of singing voice handicap and vocal fatigue.

**Methods:**

The study sample comprised of 52 professional classical choir singers, aged 31 to 72 years. They answered an online questionnaire in Google Forms, addressing their characterization, self-assessment of voice, the Voice Handicap Index-10 (VHI-10), Classical Singing Handicap Index (CSHI), and Vocal Fatigue Index (VFI).

**Results:**

The mean self-assessment of voice was between “Good” and “Very good” (1.2). The mean total VHI-10 score was 1.35, which is below the cutoff. The mean total CSHI score was 10.04. The mean total VFI score was 10.83, near the cutoff value. Classical singers who use their voice to give examples to students in their classes had higher scores in VHI-10 (p = 0.013), VFI voice restriction (p = 0.011), and VFI total score (p = 0.015). Besides, classical singers who already visited a Speech-Language Pathologist for voice problems had higher scores in VFI voice restriction (p = 0.040) and VFI recovery with voice rest (p = 0.019), in addition to correlations between instrument scores.

**Conclusion:**

Professional classical singers did not have voice handicaps. However, their self-perception of vocal fatigue was more present when the singing voice was used, such as giving examples with their own voice in class. Having had voice problems and visited a Speech-Language Pathologist in the past led to a greater perception of vocal recovery with rest.

## INTRODUCTION

Individuals who seek improvement in a school of classical singing (generally known as opera singing) aim for vocal characteristics and qualities adequate for the different types of singing encompassed in the repertoire. Thus, they acquire significant ability for the demands that typify such voices and make them appropriate for this line of singing^(1)^.

Classical singers, within their respective technical schools, need to understand, use, and appropriate breathing/phonation coordination and capacity^(2)^. Classical singing requires projected and enriched harmonics, enhanced by adjustments performed with excellence and precision – otherwise, they can lead to vocal changes, which harm the quality of life and singing handicap^(3-5)^.

These adjustments vary greatly according to the repertoire and commonly imply a total reconfiguration of the vocal tract, which is often only achieved after years of training^(5)^. In many cases, the lack of vocal warm-up and cool-down may cause strain, which in turn may overload the entire vocal tract musculature due to the high demand^(6-8)^. Compared to individuals who do not sing and make moderate use of their voice in daily speech^(1,9)^, theirs can be considered a much greater physical demand.

Singers are the occupational voice users at greatest risk of vocal complaints^(10)^, which is intensified in classical singing. Rehearsals for an opera production can be exhausting. The dynamics of rehearsals require many repetitions of musical and scenic sections for many hours and weeks to achieve the organicity of the show. In the final rehearsals and during the performances, the singer also uses costumes and props that are often uncomfortable, hot, and heavy. They usually change costumes successively according to the acts or scenes, which they are required to do in a minimum time, possibly causing more fatigue and stress. The path from the stage to the dressing room often has several flights of stairs, covered in a hurry.

Stretching helps the muscles and conditions the body for the theatrical presentation and opens the chest, contributing to the maintenance of posture^(11)^. The singer's physical body can also be required several times to build a character. Following the scenic director’s instructions, the singer physically adapts to the character’s mannerisms and emotional or stylistic intentions, which may hinder the use of the body to sing^(12)^. Therefore, classical singers also need body awareness and work to develop scenic gestures and/or postures^(13)^, as postural freedom and control favor vocal stability during interpretation. Hence, it is important to raise classical singers’ awareness of the benefits of bodywork^(11,14)^.

Lifestyle can also lead to complaints if the singer has unhealthy habits, such as smoking, alcohol consumption, and recreational drug use^(15)^. Moreover, vocal fatigue is noticeable to the singer, manifesting itself with increased phonatory effort, which improves with adequate vocal rest. Self-reported vocal fatigue can be identified with specific instruments^(16)^. However, it may also manifest as a pure condition with no specific apparent etiology or as a component of other voice disorders^(17)^. Therefore, it must be identified, and its symptoms must be discussed, to address final considerations about its biomechanical and physiological mechanism^(18)^.

In general, classical singers report having a good voice and no singing handicap, thanks to the many years of studying vocal techniques to meet the demands^(19)^. Many singers believe that the perception of vocal fatigue may be associated with dynamic aspects of the voice (e.g., tessitura), which are the most affected when professionals become vocally tired. Vocal fatigue has been demonstrated by changes in kinesthetic and proprioceptive sensations and vocal dynamics^(6)^.

Thus, singers must be able to self-evaluate, identifying possible vocal changes early^(20-22)^. Classical singers must know about and use Speech-Language Pathology care^(15)^. Likewise, their performance is perceivably better when they quickly identify any changes and seek a specialized professional^(1,4,20)^. Self-assessing singers have a unique perception, possibly not directly related to the clinician's evaluation^(23)^. Self-assessment data can verify the effectiveness of treatment and contribute to direct clinical practice^(24)^.

Therefore, this study aimed to map vocal risk in professional classical singers, analyzing their vocal self-assessment and self-perceived singing voice handicap and vocal fatigue.

## METHOD

Research approved by the Research Ethics Committee of the University of Taubaté (UNITAU) under evaluation report no. 4.541.236, of February 15, 2021.

Cross-sectional quantitative study with a convenience sample recruited from the lead researcher's contact with the participating institutions. The study included 52 classical singers of all voice types from Brazilian professional choirs, aged 31 to 72 years (mean of 49.78 years), of both sexes (28 men and 24 women). The inclusion criteria were classical singers regularly active in their choirs, who had been singing professionally for at least 1 year in their choirs, and who had online access to answer the instruments. The exclusion criteria were neurological and/or psychiatric medical diagnosis that prevented them from answering the instruments; anatomical or functional sequelae from medically diagnosed neurological, laryngeal, or oncological head and neck diseases, and history of cervical or laryngeal trauma.

The invited professional classical singers were informed about the study objectives. Those who disagreed with them could stop their participation at any time, without any personal or professional loss.

All data were collected online via Google Forms. Singers who agreed to participate responded to the informed consent form, checking the corresponding field to consent to their participation in the study, and filled out the following instruments: Vocal Characterization and Self-Assessment Questionnaire, Voice Handicap Index - 10 (VHI-10)^(21)^, Classical Singing Handicap Index (CSHI)^(19)^, and Vocal Fatigue Index (VFI)^(16)^.

They first filled out the Vocal Characterization and Self-Assessment Questionnaire, with specific information about vocal habits, daily and occupational voice use, rehearsal routine, presentations, vocal complaints, and an item with the question: “What do you think of your voice?”, whose response options were “excellent, very good, good, fair, or poor”.

VHI-10^(21)^ has 10 questions with an answer key from 0 (never) to 4 (always), whose total score is calculated by simply summing all answers, ranging from 0 to 40 points. The cutoff in VHI-10^(21)^ is 7.5 points, separating dysphonic from vocally healthy individuals, with 0.981 sensitivity and 1.000 specificity^(25)^.

CSHI^(19)^ is an exclusive vocal self-assessment instrument for classical singers. It assists Speech-Language Pathologists, choir directors, singing teachers, and vocal coaches in identifying vocal difficulties in classical singers, including those related to technical issues. It has 30 items equally divided into three subscales: Disability (10 items that address the impact of the voice problem on professional activities), Handicap (10 items that address the psychological impact of the voice problem), and Impairment (10 items that address self-perceived voice characteristics). The CSHI has an answer key ranging from 0 (never) to 4 (always), with three partial scores (Disability, Handicap, and Impairment, ranging from 0 to 40 points each) and a total score, calculated by simply summing the three partial scores (from 0 to 120 points)^(19)^.

VFI^(16)^ is a self-assessment protocol for the perception of vocal fatigue and vocal recovery with rest. Its validated Brazilian Portuguese version has 17 questions, divided into 4 factors: Factor 1 - Tiredness and voice impairment (seven items), Factor 2 - Avoidance of voice use (three items), Factor 3 - Physical discomfort (four items), and Factor 4 - Improvement of voice symptoms with rest (three items). The answer key ranges from 0 (never) to 4 (always). Partial scores for Factors 1, 2, 3, and 4 are calculated by the raw sum of the selected responses, and the total VFI score is calculated with the following formula: VFI total score = Factor 1 + Factor 2 + Factor 3 + (12 - Factor 4)^(16)^. The cutoffs of the validated Brazilian Portuguese VFI version are as follows: Factor 1 - Tiredness and voice impairment = 4.5 points; Factor 2 - Avoidance of voice use = 3.5 points; Factor 3 - Physical discomfort = 1.5 points; Factor 4 - Improvement of voice symptoms with rest = 8.5 points; and total VFI score = 11.5 points^(16)^.

The data underwent descriptive and inferential analyses using SPSS 25.0 software. The significance level was set at 5% for inferential analyses. The descriptive analysis of quantitative variables calculated the measures of central tendency (mean and median), variability (standard deviation), and position (minimum, maximum, and quartiles one and three). The descriptive analysis of qualitative variables calculated their absolute and relative percentage frequency. Quantitative variables were analyzed for normality with the Shapiro-Wilk Test and presented a non-normal distribution. The inferential analysis comparing non-normal quantitative and ordinal qualitative variables between two independent groups was performed with the Mann-Whitney Test. The correlation between non-normal quantitative and ordinal qualitative variables was performed with the Spearman Correlation Test.

## RESULTS

The study results are presented in Tables 1 to 5 below. Table 1 highlights the most significant findings in research participants regarding distribution by sex, the name of the choir in which they sing professionally, vocal classification, current singing study and regular work for more than a year, time as a professional singer, vocal warm-ups and cool-downs, vocal habits, consultation with a healthcare team, treatments for vocal problems, complaints of vocal fatigue, and vocal rest during the day. Moreover, Table 1 describes “medical diagnosis of neurological laryngeal or oncological head and neck disease or history of cervical or laryngeal trauma” as an exclusion criterion; nevertheless, an individual responded to the question as having been diagnosed with “granuloma in cartilage”. Hence, this person does not perceive themselves as having a limitation and/or restriction or sequelae that compromises and/or limits their singing.

**Table 1:** Descriptive analysis of the characterization of the sample

Variable and categories	n	%
Sex		
Males	28	53.85
Females	24	46.15
Name of the choir where you sing professionally:		
*Coral Lírico do Theatro Municipal de São Paulo*	33	63.46
*Coral Paulistano do Theatro Municipal de São Paulo*	2	3.85
*Coro da Orquestra Sinfônica do Estado de São Paulo*	4	7.69
*Coro Municipal do Rio de Janeiro*	6	11.54
*Coro da Ópera de Manaus / Coral Lírico do Amazonas*	4	7.69
*Coral / Coro Lírico de Minas Gerais*	3	5.77
Voice type:		
Soprano	13	25.00
Mezzo	6	11.54
Contralto	5	9.62
Tenor	9	17.31
Baritone	12	23.08
Bass	7	13.45
Are you currently taking singing lessons with a singing teacher?		
No	32	61.54
Yes	20	38.46
Have you been regularly studying singing for over a year?		
No	12	23.08
Yes	40	76.92
How long have you been a professional singer?		
5 to 6 years	1	1.92
7 to 8 years	1	1.92
9 to 10 years	1	1.92
> 10 years	49	94.23
How many hours do you use your singing voice in your daily routine?		
Up to 1 hour	9	17.31
1 to 2 hours/day	13	25.00
3 to 5 hours/day	26	50.00
6 or + hours/day	4	7.69
Do you use your voice in class to give examples to your students?		
Yes	26	50.00
I do not teach	26	50.00
Do you warm your voice up before singing, teaching singing classes, or rehearsing?		
No	2	3.85
Yes	50	96.15
Do you cool your voice down after singing, teaching singing classes, or rehearsing?		
No	34	65.38
Yes	18	34.62
Do you currently have any vocal complaints or voice problems?		
No	52	100.00
Yes	0	0
Do you smoke any cigarettes or drugs?		
No	46	88.46
Ex-smoker	4	7.69
Yes	2	3.85
Do you drink alcoholic beverages?		
No	35	67.31
Yes	17	32.69
Have you ever visited a Speech-Language Pathologist for voice problems?		
No	24	46.15
Yes	28	53.85
Have you ever visited an otorhinolaryngologist for voice problems?		
No	13	25.00
Yes	39	75.00
Have you ever had vocal treatment? Speech-Language Pathology exercises?		
No	30	57.69
Yes	22	42.31
Singing exercises		
No	43	82.69
Yes	9	17.31
Medications		
No	31	59.62
Yes	21	40.38
Surgery		
No	51	98.08
Yes	1	1.92
Others		
No	51	98.08
Yes	1	1.92
Do you have or have you ever had a medical diagnosis of laryngeal neurological or oncological head and neck disease or a history of cervical or laryngeal trauma with anatomical or functional sequelae?		
No	51	98.08
Yes	1	1.92
Do you know anything about vocal fatigue and its causes and effects on the voice?		
No	3	5.77
Yes	49	94.23
Do you know anything about vocal rest during the day and its benefits?		
No	2	3.85
Yes	50	96.15
Do you rest your voice during the day?		
No	6	11.54
Up to 1 hour/day	7	13.46
1 to 2 hours/day	19	36.54
3 to 5 hours/day	12	23.08
6 or more hours/day	8	15.38
Do you know anything about vocal health and hygiene and their benefits to the voice?		
No	3	5.77
Yes	49	94.23

Descriptive analysis

**Caption:** n = absolute frequency; % = relative frequency

**Table 5:** Correlation between vocal self-assessment, VHI-10, CSHI, and VFI in classical singers

		CSHI Disability	CSHI Handicap	CSHI Impairment	CSHI Total	VFI Tiredness and voice impairment	VFI Avoidance of voice use	VFI Physical discomfort	VFI Improvement of voice symptoms with rest	VFI Total
VHI-10 Total	r	0.387	0.422	0.356	0.442	0.415	0.218	0.241	-0.051	0.339
	p-value	0.005*	0.002*	0.010*	0.001*	0.002*	0.120	0.086	0.721	0.014*
CSHI Disability	r		0.450	0.716	0.868	0.329	0.133	0.289	0.129	0.149
	p-value		0.001*	0.000*	0.000*	0.017*	0.349	0.038*	0.362	0.293
CSHI Handicap	r			0.539	0.693	0.314	0.366	0.255	0.311	0.076
	p-value			0.000*	0.000*	0.023*	0.008*	0.069	0.025*	0.594
CSHI Impairment	r				0.921	0.378	0.142	0.400	0.193	0.127
	p-value				0.000*	0.006*	0.315	0.003*	0.170	0.371
CSHI Total	r					0.410	0.240	0.373	0.218	0.160
	p-value					0.003*	0.086	0.007*	0.121	0.258
VFI Tiredness and voice impairment	r						0.511	0.472	0.153	0.606
	p-value						0.000*	0.000*	0.280	0.000*
VFI Avoidance of voice use	r							0.353	0.287	0.528
	p-value							0.010*	0.039*	0.000*
VFI Physical discomfort	r								0.236	0.369
	p-value								0.091	0.007*
VFI Improvement of voice symptoms with rest	r									-0.481
	p-value									0.000*

* Significant values (p ≤ 0.05) – Spearman correlation test

**Caption:** r = correlation coefficient; VHI-10 = Voice Handicap Index-10; CSHI = Classical Singing Handicap Index; VFI = Vocal Fatigue Index

Table 2 presents the descriptive analysis of mean, standard deviation, minimum and maximum values, quartiles one and three, and median of the dependent variables – i.e., the classical singers’ self-assessment: vocal self-assessment, VHI-10 total score, CSHI partial and total scores, and VFI partial and total scores.

**Table 2:** Descriptive analysis of vocal self-assessment, VHI-10, CSHI, and VFI in classical singers

Variable	Mean	SD	Minimum	Maximum	Q_1_	Median	Q_3_
Vocal self-assessment	1.10	0.75	0.00	3.00	1.00	1.00	1.75
VHI-10 Total	1.35	1.77	0.00	7.00	0.00	1.00	2.00
CSHI Disability	3.44	3.20	0.00	14.00	0.00	3.00	5.75
CSHI Handicap	2.35	2.92	0.00	12.00	0.00	1.00	4.00
CSHI Impairment	4.25	4.18	0.00	16.00	0.25	3.00	6.00
CSHI Total	10.04	9.19	0.00	41.00	3.25	8.00	12.00
VFI Tiredness and voice impairment	2.52	2.75	0.00	12.00	0.00	2.00	4.00
VFI Avoidance of voice use	3.42	2.94	0.00	10.00	1.00	3.00	6.00
VFI Physical discomfort	0.63	1.07	0.00	4.00	0.00	0.00	1.00
VFI Improvement of voice symptoms with rest	7.75	4.21	0.00	12.00	4.00	9.00	12.00
VFI Total	10.83	5.85	0.00	25.00	8.00	11.00	15.00

Descriptive analysis

**Caption:** n = absolute frequency; % = relative frequency; SD = standard deviation; Q_1_ = quartile one; Q_3_ = quartile three; VHI-10 = Voice Handicap Index-10; CSHI = Classical Singing Handicap Index; VFI = Vocal Fatigue Index

Table 3 shows that classical singers who give examples to their students in class using their own voices have higher scores on VHI-10 (p = 0.013), VFI Avoidance of voice use (p = 0.011), and VFI total score (p = 0.015).

**Table 3:** Inferential comparison analysis between vocal self-assessment, VHI-10, CSHI, and VFI in relation to the variable, “Do you use your voice in class to give examples to your students?” in classical singers

Variable	Do you use your voice in class to give examples to your students?	Mean	SD	Minimum	Maximum	Q_1_	Median	Q_3_	p-value
Vocal self-assessment	Yes	1.08	0.69	0.00	3.00	1.00	1.00	1.00	0.831
	I do not teach	1.12	0.82	0.00	3.00	0.75	1.00	2.00	
VHI-10 Total	Yes	1.88	1.99	0.00	7.00	0.00	1.00	2.25	0.013*
	I do not teach	0.81	1.36	0.00	5.00	0.00	0.00	2.00	
CSHI Disability	Yes	3.73	3.01	0.00	11.00	1.75	3.00	6.25	0.368
	I do not teach	3.15	3.41	0.00	14.00	0.00	3.00	5.25	
CSHI Handicap	Yes	2.73	2.79	0.00	10.00	1.00	1.00	4.25	0.066
	I do not teach	1.96	3.05	0.00	12.00	0.00	0.00	3.25	
CSHI Impairment	Yes	3.85	3.40	0.00	13.00	1.00	3.00	6.00	0.875
	I do not teach	4.65	4.87	0.00	16.00	0.00	3.00	7.25	
CSHI Total	Yes	10.31	8.03	1.00	31.00	4.00	8.00	12.25	0.463
	I do not teach	9.77	10.37	0.00	41.00	0.00	8.50	13.25	
VFI Tiredness and voice impairment	Yes	2.88	3.17	0.00	12.00	0.00	2.00	6.00	0.553
	I do not teach	2.15	2.27	0.00	7.00	0.00	2.00	3.25	
VFI Avoidance of voice use	Yes	4.42	2.83	0.00	10.00	1.75	5.00	6.25	0.011*
	I do not teach	2.42	2.74	0.00	10.00	0.00	1.50	4.00	
VFI Physical discomfort	Yes	0.69	1.16	0.00	4.00	0.00	0.00	1.00	0.665
	I do not teach	0.58	0.99	0.00	3.00	0.00	0.00	1.00	
VFI Improvement of voice symptoms with rest	Yes	7.31	3.86	0.00	12.00	4.00	8.00	12.00	0.344
	I do not teach	8.19	4.58	0.00	12.00	3.75	11.00	12.00	
VFI Total	Yes	12.69	6.29	0.00	25.00	9.50	12.50	16.25	0.015*
	I do not teach	8.96	4.79	0.00	20.00	5.00	9.00	12.00	

* Significant values (p ≤ 0.05) – Mann-Whitney test

**Caption:** SD = standard deviation; Q_1_ = quartile one; Q_3_ = quartile three; VHI-10 = Voice Handicap Index-10; CSHI = Classical Singing Handicap Index; VFI = Vocal Fatigue Index

Table 4 shows that classical singers who perform vocal cool-down after singing, giving singing lessons, or rehearsing have higher VFI vocal restriction scores than those who do none of these (p = 0.016).

**Table 4:** Inferential comparison analysis between vocal self-assessment, VHI-10, CSHI, and VFI in relation to the variable, “Do you cool your voice down after singing, teaching singing classes, or rehearsing?” in classical singers

Variable	Do you cool your voice down?	Mean	SD	Minimum	Maximum	Q_1_	Median	Q_3_	p-value
Vocal self-assessment	No	1.09	0.75	0.00	3.00	1.00	1.00	2.00	0.983
	Yes	1.11	0.76	0.00	3.00	1.00	1.00	1.25	
VHI-10 Total	No	1.35	2.00	0.00	7.00	0.00	0.00	2.00	0.322
	Yes	1.33	1.28	0.00	5.00	0.00	1.00	2.00	
CSHI Disability	No	3.26	3.23	0.00	14.00	0.00	3.00	5.00	0.559
	Yes	3.78	3.21	0.00	11.00	1.75	3.00	6.25	
CSHI Handicap	No	2.15	2.95	0.00	12.00	0.00	1.00	4.00	0.276
	Yes	2.72	2.93	0.00	10.00	0.75	1.50	4.25	
CSHI Impairment	No	4.44	4.54	0.00	16.00	0.00	4.00	6.25	0.808
	Yes	3.89	3.50	0.00	13.00	2.00	3.00	5.50	
CSHI Total	No	9.85	9.61	0.00	41.00	2.50	8.00	12.75	0.664
	Yes	10.39	8.58	2.00	31.00	4.00	7.50	12.25	
VFI Tiredness and voice impairment	No	2.38	2.66	0.00	12.00	0.00	2.00	3.00	0.729
	Yes	2.78	2.98	0.00	8.00	0.00	1.50	6.00	
VFI Avoidance of voice use	No	2.71	2.68	0.00	10.00	0.00	2.00	5.00	0.016*
	Yes	4.78	3.00	0.00	10.00	1.75	5.50	7.00	
VFI Physical discomfort	No	0.56	0.99	0.00	4.00	0.00	0.00	1.00	0.562
	Yes	0.78	1.22	0.00	4.00	0.00	0.00	1.25	
VFI Improvement of voice symptoms with rest	No	7.12	4.37	0.00	12.00	3.75	8.50	12.00	0.122
	Yes	8.94	3.72	1.00	12.00	6.75	10.50	12.00	
VFI Total	No	10.53	5.32	0.00	25.00	7.75	11.00	14.25	0.623
	Yes	11.39	6.87	0.00	23.00	7.25	10.50	17.00	

* Significant values (p ≤ 0.05) – Mann-Whitney test

**Caption:** SD = standard deviation; Q_1_ = quartile one; Q_3_ = quartile three; VHI-10 = Voice Handicap Index-10; CSHI = Classical Singing Handicap Index; VFI = Vocal Fatigue Index**.**

Table 5 compares the various significant correlations of varying strengths between protocols. The strongest ones are between CSHI total and disability scores (r = 0.868; p = 0.000) and CSHI total and impairment scores (r = 0.921; p = 0.000*). The analysis between scores from different protocols found that the strongest significant correlations occurred between VFI Tiredness and voice impairment and VHI-10 total score (r = 0.415; p = 0.002*) and between VFI Tiredness and voice impairment and CSHI total score (r = 0.410; p = 0.003*). This indicates a possible relationship between self-perceived fatigue and vocal limitation and self-reported general and specific singing voice handicaps.

## DISCUSSION

Classical singing is a style within the art of singing that requires absolute control of emotions and precise control of the entire phonatory system^(11)^. Such harmonious awareness not only provides a rich vocal interpretation, which we call technique, but also fills the audience and performers with pleasure and, consequently, quality of life^(23)^.

Singers are described as the elite of occupational voice users, like actors and teachers, making up a social group whose voices are the main tools of their trade – hence, vocal fatigue is a topic common to them^(18)^. Studies indicate that any slight change perceived by this community can have a major impact on their quality of life^(19)^.

The present study aimed to identify how classical singers from professional Brazilian choirs perceive their voices, vocal fatigue, and recovery with vocal rest, using internationally recognized psychometric instruments^(24)^ validated and/or with cultural equivalence in Brazil^(16,19,21)^.

Cultural equivalence has already proved to be efficient for research on instruments translated into other languages^(26-29)^, ensuring reliable results that can be reproduced with statistical sensitivity. This makes them significant for these professionals’ daily vocal demands^(24)^.

Thus, the importance of self-assessment^(20)^ is that it contributes to robust clinical findings and effectiveness in choosing the best therapeutic approach^(21,22)^.

It can be inferred from Table 1 in this study that the research participants have technical-vocal maturity. They do not report feeling voice handicaps^(19)^ thanks to either the years of singing lessons (76.92%) or the experience acquired over time with their practice and demands (94.23%)^(19)^. Thus, they agree with the literature in that they consider their voices good to very good^(21)^.

The literature also points out that these professionals, with years of technical study and daily experience, have a greater perception of themselves^(15)^. Although it is not the aim of the study to address any predominance related to sex and/or vocal register, the characterization questionnaire (Table 1) highlights a greater participation of sopranos (n = 13; 25%), baritones (n = 12; 23.08%), and tenors (n = 9; 17.31%).

The literature describes that classical singers’ vocal adjustments take a complex mechanism involving not only vocal tract adaptation (preparing it for the emission of a musical note relevant to each register)^(13)^ but also the breathing/phonation coordination capacity (using the tension generated in the chest to adjust the subglottic pressure and control the passage of the air column, providing the performer's voice with rich harmonics and projection). It can be stated that singers are required to have articulation to ensure an intelligible sung text, almost always in a language other than the individual's fluent linguistic domain. Over articulation is often needed, depending on the height of the musical notation in which this text is used. Lips, tongue position, and jaw movement/opening cannot be restricted and constitute an articulatory adjustment for the efficiency and intelligibility of certain phonemes in another language, in addition to the refinement of singing. There is, therefore, a set of powers (pulmonary, phonatory, and articulatory) that need to work together and harmoniously. Any slight inaccuracy/incoordination in carrying out these adjustments can lead the professional to perceive a compromised performance^(1,2)^.

The present study found correlations between the results of some instruments that indicate the participants’ perception of fatigue. The results in Table 2 show that they do not perceive themselves as having voice handicaps (VHI-10: 1.35; CSHI: 2.35)^(4)^. However, there is a correlation with those who perceived disability (3.44) and/or impairment (4.25) in CSHI (whose total score in this study was 10.04)^(30)^ and VFI Tiredness and voice impairment: 2.52 (the highest score in this item was 4.5) and VFI Avoidance of voice use: 3.42 (the highest score in this item was 3.5)^(16)^.

Table 3 presents the results for individuals who, in addition to their daily routine, are singing teachers and use their voices to give examples to students in class. The p-values show proximity between item scores that indicate restriction (VHI-10: 1.88; p = 0.06; VFI Avoidance of voice use: 4.42; p = 0.011). The VFI total score for individuals who are teachers and give examples to students is 12.69 (p = 0.015) – i.e., above the total score for the protocol, whose cutoff was set at 11.5^(16)^.

This study also found relevant results of the perception of improvement of voice symptoms with rest (VFI: 10.69; p = 0.015), as 53.85% of these individuals reported seeking help from Speech-Language Pathologists due to voice problems, and 75% have already sought treatment from an otorhinolaryngologist^(15)^.

Vocal fatigue may be associated with vocal hyperfunction. It is a symptom almost always reported as vocal effort and discomfort, reduced pitch range and flexibility, reduced vocal projection or power, reduced voice quality control, increased speech symptoms throughout the day, and improvement after rest. The results in Table 4 show that 54% of singers who perform vocal cool-down after singing and/or teaching singing classes in which they give examples to students with their own voice have higher VFI restriction scores (p = 0.016)^(16,17)^. Although the literature described vocal cool-down as the adjustment that allows muscles to return to a colloquial voice pattern, there is no consensus on its effects in other practices such as sports medicine, physical therapy, or physical education. Vocal cool-down is believed to help dissipate muscle residues, especially lactic acid, with a reduction in post-exercise muscle pain for 24 to 48 hours. Thus, the study participants’ self-perception is that vocal cool-down, even after singing activity/singing lessons, did not provide their voices with an effective and immediate feeling of comfort, as they were previously hyperfunctioning.

As a limitation, the present study was collected during the COVID-19 pandemic when singers were in lockdown – i.e., they were not daily rehearsing and/or teaching in-person classes; rather, they maintained online work activities.

## CONCLUSION

Professional classical singers in this study did not have singing voice handicap. However, issues related to self-perceived vocal fatigue are more present when associated with activities other than the use of the singing voice, such as giving examples in voice classes. Having had vocal problems and visited Speech-Language Pathologists in the past led to a greater perception of vocal recovery with rest.
