# Supplementary material for: Vocal risk mapping in classical singers: an analysis of the self-perception of voice quality, vocal fatigue, and singing voice handicap
Source: Codas. 2024 Jun 14;36(4):e20230088. doi: 10.1590/2317-1782/20242023088en (PMC11189155; doi:10.1590/2317-1782/20242023088en)
Supplement: Translation

# Translation: Mapeamento do risco vocal em cantores eruditos: análise da autopercepção da qualidade da voz, fadiga vocal e desvantagem no canto

**DOI:** 10.1590/2317-1782/20242023088pt

**Authors:** Diógenes Gomes, Felipe Moreti, Mara Behlau

**Affiliations:** 1 Centro de Estudos da Voz - CEV - São Paulo (SP), Brasil.; 2 Departamento de Fonoaudiologia, Faculdade de Filosofia e Ciências, Universidade Estadual Paulista “Júlio de Mesquita Filho” - Unesp - Marília (SP), Brasil.

**Keywords:** Voz, Canto, Autoteste, Inquéritos e Questionários, Fadiga, Fonoaudiologia

## Abstract

**Objetivo:**

Mapear o risco vocal em cantores eruditos profissionais, analisando sua autoavaliação vocal e autopercepção de desvantagem vocal no canto e fadiga vocal.

**Método:**

Participaram deste estudo 52 cantores eruditos de coros profissionais, entre 31 e 72 anos, que preencheram online (Formulários Google) um questionário de caracterização, autoavaliação vocal, bem como os instrumentos Índice de Desvantagem Vocal 10 - IDV-10, Índice de Desvantagem para o Canto Clássico - IDCC e Índice de Fadiga Vocal - IFV.

**Resultados:**

A média da autoavaliação vocal ficou entre “Boa” e “Muito boa” (1,2). A média do escore total do IDV-10 foi 1,35, abaixo da nota de corte. A média do escore total do IDCC foi 10,04. A média do escore total do IFV foi de 10,83, próxima da nota de corte. Cantores eruditos que, em suas aulas, dão exemplos aos alunos com a própria voz apresentam maiores escores nos instrumentos IDV-10 (p=0,013), IFV restrição vocal (p=0,011) e IFV escore total (p=0,015). Cantores eruditos que já procuraram fonoaudiólogo devido a problemas de voz apresentam maiores escores no IFV fator restrição vocal (p=0,040) e no IFV fator recuperação com repouso vocal (p=0,019), além de terem correlações entre os escores dos instrumentos.

**Conclusão:**

Cantores eruditos profissionais não apresentam desvantagem vocal. Porém, questões relacionadas à autopercepção de fadiga vocal se mostram mais presentes quando relacionadas às atividades de uso da voz cantada, como dar exemplos em aulas com a própria voz. Ter tido problema vocal e procurado fonoaudiólogo no passado proporciona maior percepção de recuperação vocal com repouso.

## INTRODUÇÃO

O indivíduo que se aperfeiçoa numa escola de canto erudito, ou canto lírico, como é geralmente chamado, está em busca de uma característica vocal e de qualidade apropriadas para as diversas modalidades de canto que abrangem o repertório, dotando de significativa habilidade para as exigências que tipificam esta voz e a tornam apropriada para a execução dessa linha de canto^(1)^.

Cantores eruditos, dentro de suas respectivas escolas técnicas, necessitam da compreensão, do uso e adequação da coordenação e capacidade pneumofonoarticulatória^(2)^. No canto erudito, a necessidade de projeção e enriquecimento de harmônicos é valorizada com o uso de ajustes que precisam ser muito bem executados e de forma correta, do contrário podem favorecer o aparecimento de alterações vocais, as quais prejudicam a qualidade de vida e resultam em desvantagem no canto^(3-5)^.

Estes ajustes variam muito de acordo com o repertório exigido e comumente implica numa total reconfiguração do trato vocal, o que muitas vezes só se alcança após anos de treinamento^(5)^. Pode haver, em muitos casos, um estresse relacionado com a falta da prática de aquecimento e desaquecimento vocal, o que, devido à alta demanda gerada, pode ocasionar uma sobrecarga em toda a musculatura do aparelho fonador^(6-8)^. Em comparação com indivíduos que não cantam e fazem uso moderado da voz na fala diária^(1,9)^, é possível considerar essa como uma exigência física muito maior.

Cantores são os profissionais da voz com maior risco de apresentar queixa vocal^(10)^, o que se intensifica no cenário do canto erudito. Os ensaios para a produção de uma ópera podem ser extenuantes. A dinâmica dos ensaios requer muitas repetições dos trechos musicais e cênicos, durante horas, inclusive semanas, para se conseguir a organicidade do espetáculo. Nos ensaios finais e durante as récitas, o cantor usa, ainda, figurinos e adereços muitas vezes desconfortáveis, quentes e pesados. Em geral há trocas sucessivas desse figurino para determinado ato ou cena, sendo solicitado que o cantor o faça num tempo mínimo, podendo gerar mais cansaço e estresse. O trajeto do palco até o camarim muitas vezes é feito correndo e utilizando vários lances de escada.

A prática de alongamento favorece a musculatura para condicionar o corpo à parte teatral e propicia abertura do tórax, contribuindo para a manutenção da postura^(11)^. O corpo físico do cantor também pode ser exigido diversas vezes na busca da construção de uma personagem. Para atender à solicitação do diretor cênico, o cantor adapta-se fisicamente na elaboração de trejeitos e intenções emocionais ou estilísticas à personagem que podem atrapalhar o corpo que canta^(12)^. Portanto, cantores eruditos também necessitam de consciência e trabalho corporal na elaboração de um gestual e/ou postura cênica^(13)^, pois liberdade e controle da postura favorecem a estabilidade vocal durante a interpretação. Nesse sentido, é importante promover a conscientização dos benefícios do trabalho corporal a um cantor lírico^(11,14)^.

O estilo de vida também pode favorecer o aparecimento de queixas, se o cantor tem hábitos não saudáveis, como tabagismo, consumo de álcool e uso de drogas recreativas^(15)^. Além disso, a fadiga vocal é perceptível ao cantor, manifestando-se com o aumento do esforço fonatório, o qual obtém melhora com repouso vocal adequado. A fadiga vocal autorreferida pode ser identificada por meio de instrumentos específicos^(16)^. No entanto, pode também se apresentar como uma condição pura, de modo que nenhuma etiologia específica seja aparente ou mesmo componente de outros distúrbios da voz^(17)^. Isso torna necessária a sua identificação, bem como a discussão sobre seus sintomas, para que então as considerações finais sobre seu mecanismo biomecânico e fisiológico sejam trabalhadas^(18)^.

Em geral os cantores eruditos relatam ter boa voz e não referem desvantagem no canto, o que está relacionado aos muitos anos de estudo de técnica vocal para atenderem a exigência de demandas^(19)^. Sobre a percepção de fadiga vocal, muitos cantores acreditam que ela pode estar associada aos aspectos dinâmicos da voz (por exemplo, a tessitura), os quais são considerados como os mais afetados quando os profissionais se cansam vocalmente. A fadiga vocal foi comprovada por alterações nas sensações cinestésicas, proprioceptivas e na dinâmica vocal^(6)^.

Logo, é de extrema importância que o cantor possa ter a habilidade de se autoavaliar, identificando de modo precoce possíveis alterações vocais^(20-22)^. É essencial que cantores líricos conheçam e recorram a atendimento fonoaudiológico^(15).^ Do mesmo modo, é notório que seu desempenho é melhor quando estes identificam rapidamente alguma alteração, recorrendo a um profissional especializado^(1,4,20)^. Ao se avaliar, o cantor tem uma percepção única, a qual, inclusive, pode não estar diretamente relacionada à avaliação do clínico^(23)^. Os dados de autoavaliação de um indivíduo são instrumentos capazes de verificar a eficácia de um tratamento e contribuir para que a prática na clínica seja direta^(24)^.

Assim sendo, o objetivo deste estudo é um mapeamento do risco vocal em cantores eruditos profissionais, analisando sua autoavaliação vocal e autopercepção de desvantagem vocal no canto e fadiga vocal.

## MÉTODO

Pesquisa aprovada pelo Comitê de Ética em Pesquisa - CEP da Universidade de Taubaté - UNITAU, parecer nº 4.541.236, de 15 de fevereiro de 2021.

Estudo transversal e quantitativo, com amostra de conveniência, coletada a partir do contato do pesquisador principal com as instituições participantes. Foram incluídos neste estudo 52 cantores eruditos de coros profissionais brasileiros, de todos os naipes vocais com idades entre 31 e 72 anos (média 49,78 anos), de ambos os gêneros (28 homens e 24 mulheres). Os critérios de inclusão foram: cantores eruditos regularmente ativos nos respectivos coros; prática de canto profissional há no mínimo um ano nos respectivos coros e acesso online para o preenchimento dos instrumentos. Já os critérios de exclusão foram: diagnóstico médico neurológico e/ou psiquiátrico que impedissem o preenchimento dos instrumentos; sequelas anatômicas ou funcionais por diagnóstico médico de doenças neurológicas laríngeas ou oncológicas em região de cabeça e pescoço além de história de traumas cervicais ou laríngeos.

Os cantores eruditos profissionais convidados foram informados sobre os objetivos deste estudo. Caso não concordassem, poderiam interromper suas participações a qualquer momento, sem nenhum tipo de ônus pessoal ou profissional.

Toda a coleta foi realizada online por meio de Formulários Google. Ao aceitarem participar, os cantores responderam o termo de consentimento livre e esclarecido, assinalando o campo correspondente de forma a assentir sua participação no estudo, além do preenchimento dos instrumentos: Questionário de Caracterização e Autoavaliação Vocal, Índice de Desvantagem Vocal 10 - IDV-10^(21)^, Índice de Desvantagem para o Canto Clássico - IDCC^(19)^ e Índice de Fadiga Vocal - IFV^(16)^.

O Questionário de Caracterização e Autoavaliação vocal foi o primeiro instrumento a ser preenchido. Ele contém informações específicas sobre hábitos vocais, uso da voz cotidiana e profissionalmente, rotina de ensaios, apresentações, queixa vocal e um item com a pergunta: “O que você acha da sua voz?”, cujas opções de respostas eram “excelente, muito boa, boa, razoável ou ruim”.

O Índice de Desvantagem Vocal 10 - IDV-10^(21)^ possui 10 questões com chave de respostas de 0 (nunca) a 4 (sempre), cujo cálculo de escore total se faz pela soma simples de todas as respostas assinaladas, variando de 0 a 40 pontos. O IDV-10^(21)^ tem uma nota de corte de 7,5 pontos, a qual separa indivíduos disfônicos daqueles vocalmente saudáveis, com sensibilidade de 0,981 e especificidade de 1,000^(25)^.

O Índice de Desvantagem para o Canto Clássico - IDCC^(19)^ é um instrumento de autoavaliação vocal exclusivo para cantores eruditos. Ele auxilia fonoaudiólogos, regentes de coros, professores de canto e preparadores vocais na identificação de dificuldades vocais dos cantores clássicos, incluindo dificuldades por questões técnicas. O instrumento apresenta 30 itens, divididos igualmente em 3 subescalas: Incapacidade (10 itens que abordam o impacto do problema de voz nas atividades profissionais), Desvantagem (10 itens que abordam o impacto psicológico do problema de voz) e Defeito (10 itens que abordam a autopercepção das características da voz). O IDCC tem chave de respostas que varia de 0 nunca a 4 sempre, com três escores parciais (Incapacidade, Desvantagem e Defeito, variando de 0 a 40 pontos cada escore) e um escore total, calculado pela soma simples dos três escores parciais (de 0 a 120 pontos)^(19)^.

O Índice de Fadiga Vocal - IFV^(16)^ é um protocolo de autoavaliação da percepção de fadiga vocal e recuperação vocal com repouso. Sua validação no português brasileiro possui 17 questões, divididas em 4 fatores: fator 1 - Fadiga e limitação vocal (sete itens), Fator 2 - Restrição vocal (três itens), Fator 3 - Desconforto físico associado à voz (quatro itens) e Fator 4 - Recuperação com repouso vocal (três itens), com chave de respostas de 0 (nunca) a 4 (sempre). O cálculo dos escores parciais dos Fatores 1, 2, 3 e 4 é realizado por meio da soma bruta das respostas assinaladas e o escore total do IFV é calculado pela fórmula a seguir: Escore total IFV = Fator 1 + Fator 2 + Fator 3 + (12 - Fator 4)^(16)^. Os valores de corte da versão validada para o português brasileiro do IFV são: Fator 1 - Fadiga e limitação vocal = 4,5 pontos; Fator 2 - Restrição vocal = 3,5 pontos; Fator 3 - Desconforto físico associado à voz = 1,5 ponto; Fator 4 - Recuperação com repouso = 8,5 pontos e Escore total do IFV = 11,5 pontos^(16)^.

Os dados foram analisados de forma descritiva e inferencial utilizando-se o software SPSS 25.0. Foi considerado um nível de significância de 5% para as análises inferenciais. Na análise descritiva das variáveis quantitativas foram calculadas as medidas de tendência central (média e mediana), variabilidade (desvio-padrão) e posição (mínimo, máximo, primeiro e terceiro quartis). Na análise descritiva das variáveis qualitativas foram calculadas a frequência absoluta e a frequência relativa percentual. As variáveis quantitativas foram a análise de normalidade com o Teste de Shapiro-Wilk e apresentaram distribuição não-normal. A análise inferencial de comparação das variáveis quantitativas não-normais e qualitativas ordinais entre dois grupos independentes foi realizada com o Teste de Mann-Whitney. A correlação entre as variáveis quantitativas não-normais e qualitativas ordinais foi realizada com o Teste de Correlação de Spearman.

## RESULTADOS

Os resultados do estudo estão apresentados nas Tabelas de 1 a 5 a seguir. Na Tabela 1 destacam-se os achados mais expressivos nos sujeitos participantes da pesquisa referente à distribuição por gênero, ao nome do coro em que cantam profissionalmente, à classificação vocal, ao estudo de canto atual e trabalho regular há mais de um ano, tempo como cantor profissional, além de realização de aquecimento e desaquecimento vocal, hábitos vocais, consulta com equipe de saúde e tratamentos para problemas vocais, queixa de fadiga vocal e repouso vocal durante o dia. Embora descrito na Tabela 1 como critério de exclusão “o indivíduo ter recebido diagnóstico médico de doenças neurológicas laríngeas ou oncológicas em região de cabeça e pescoço, ou ainda, história de traumas cervicais ou laríngeos”, o indivíduo respondente à questão relatou ter sido diagnosticado com “granuloma em cartilagem”. Este indivíduo, portanto, não se autopercebe com limitação e/ou restrição ou sequela que comprometa e/ou limite-o na função do canto.

**Tabela 1:** Análise descritiva de caracterização da amostra

Variável e categorias	n	%
Gênero		
Masculino	28	53,85
Feminino	24	46,15
Nome do coro onde você canta profissionalmente:		
Coral Lírico do Theatro Municipal de São Paulo	33	63,46
Coral Paulistano do Theatro Municipal de São Paulo	2	3,85
Coro da Orquestra Sinfônica do Estado de São Paulo	4	7,69
Coro Municipal do Rio de Janeiro	6	11,54
Coro da Ópera de Manaus / Coral Lírico do Amazonas	4	7,69
Coral / Coro Lírico de Minas Gerais	3	5,77
Classificação vocal:		
Soprano	13	25,00
Mezzo	6	11,54
Contralto	5	9,62
Tenor	9	17,31
Barítono	12	23,08
Baixo	7	13,45
Estuda canto atualmente com professor de canto?		
Não	32	61,54
Sim	20	38,46
Estuda regularmente canto há mais de um ano?		
Não	12	23,08
Sim	40	76,92
Há quanto tempo é cantor profissional?		
5 e 6 anos	1	1,92
7 e 8 anos	1	1,92
9 e 10 anos	1	1,92
>10 anos	49	94,23
Em sua rotina diária, por quantas horas você faz uso da voz cantada?		
Até 1h	9	17,31
1 e 2h/dia	13	25,00
3 a 5h/dia	26	50,00
6 ou + h/dia	4	7,69
Em suas aulas, você dá exemplo aos alunos com sua própria voz?		
Sim	26	50,00
Não dou aula	26	50,00
Realiza aquecimento vocal antes de cantar, ministrar aulas de canto ou ensaiar canto?		
Não	2	3,85
Sim	50	96,15
Realiza desaquecimento vocal depois de cantar, ministrar aulas de canto ou ensaiar canto?		
Não	34	65,38
Sim	18	34,62
Atualmente, possui alguma queixa vocal ou problema de voz?		
Não	52	100,00
Sim	0	0
Faz uso de cigarros ou drogas fumadas?		
Não	46	88,46
Ex-tabagista	4	7,69
Sim	2	3,85
Faz uso de bebidas alcoólicas?		
Não	35	67,31
Sim	17	32,69
Já procurou fonoaudiólogo por problemas de voz?		
Não	24	46,15
Sim	28	53,85
Já procurou médico otorrinolaringologista por problemas de voz?		
Não	13	25,00
Sim	39	75,00
Já realizou algum tratamento vocal? Exercícios fonoaudiológicos?		
Não	30	57,69
Sim	22	42,31
Exercícios de canto		
Não	43	82,69
Sim	9	17,31
Remédios		
Não	31	59,62
Sim	21	40,38
Cirurgia		
Não	51	98,08
Sim	1	1,92
Outros		
Não	51	98,08
Sim	1	1,92
Já teve ou tem diagnóstico médico de doenças neurológicas laríngeas ou oncológicas em região de cabeça e pescoço ou ainda história de traumas cervicais ou laríngeos que deixaram alguma sequela anatômica ou funcional?		
Não	51	98,08
Sim	1	1,92
Tem conhecimento sobre fadiga vocal, suas causas e efeitos na voz?		
Não	3	5,77
Sim	49	94,23
Tem conhecimento sobre repouso vocal durante o dia e seus benefícios?		
Não	2	3,85
Sim	50	96,15
Realiza repouso vocal durante o dia?		
Não	6	11,54
Até 1h/dia	7	13,46
1 a 2h/dia	19	36,54
3 a 5h/dia	12	23,08
6h ou mais/dia	8	15,38
Tem conhecimento sobre saúde e higiene vocal e seus benefícios na voz?		
Não	3	5,77
Sim	49	94,23

Análise descritiva

**Legenda:** n = frequência absoluta; % = frequência relativa

**Tabela 5:** Correlação entre as variáveis autoavaliação vocal, IDV-10, IDCC e IFV em cantores eruditos

		IDCC Incapacidade	IDCC Desvantagem	IDCC Defeito	IDCC Total	IFV Fadiga e limitação vocal	IFV Restrição vocal	IFV Desconforto físico associado à voz	IFV Recuperação com repouso vocal	IFV Total
IDV-10 Total	r	0,387	0,422	0,356	0,442	0,415	0,218	0,241	-0,051	0,339
	p-valor	0,005*	0,002*	0,010*	0,001*	0,002*	0,120	0,086	0,721	0,014*
IDCC Incapacidade	r		0,450	0,716	0,868	0,329	0,133	0,289	0,129	0,149
	p-valor		0,001*	0,000*	0,000*	0,017*	0,349	0,038*	0,362	0,293
IDCC Desvantagem	r			0,539	0,693	0,314	0,366	0,255	0,311	0,076
	p-valor			0,000*	0,000*	0,023*	0,008*	0,069	0,025*	0,594
IDCC Defeito	r				0,921	0,378	0,142	0,400	0,193	0,127
	p-valor				0,000*	0,006*	0,315	0,003*	0,170	0,371
IDCC Total	r					0,410	0,240	0,373	0,218	0,160
	p-valor					0,003*	0,086	0,007*	0,121	0,258
IFV Fadiga e limitação vocal	r						0,511	0,472	0,153	0,606
	p-valor						0,000*	0,000*	0,280	0,000*
IFV Restrição vocal	r							0,353	0,287	0,528
	p-valor							0,010*	0,039*	0,000*
IFV Desconforto físico associado à voz	r								0,236	0,369
	p-valor								0,091	0,007*
IFV Recuperação com repouso vocal	r									-0,481
	p-valor									0,000*

* Valores significativos (p≤0,05) - Teste de Correlação de Spearman

**Legenda:** r = coeficiente de correlação; IDV-10 = Índice de Desvantagem Vocal 10; IDCC = Índice de Desvantagem para o Canto Clássico; IFV = Índice de Fadiga Vocal

Na Tabela 2 apresenta a análise descritiva de média, desvio padrão, valores mínimo e máximo, além do 1º quartil, mediana e 3º quartil das variáveis dependentes do estudo, as quais se referem à autoavaliação vocal de cantores eruditos: autoavaliação vocal, escore total do IDV-10, escores parciais e total do IDCC, escores parciais e total do IFV.

**Tabela 2:** Análise descritiva das variáveis autoavaliação vocal, IDV-10, IDCC e IFV em cantores eruditos

Variável	Média	DP	Mínimo	Máximo	1Q	Mediana	3Q
Autoavaliação vocal	1,10	0,75	0,00	3,00	1,00	1,00	1,75
IDV-10 Total	1,35	1,77	0,00	7,00	0,00	1,00	2,00
IDCC Incapacidade	3,44	3,20	0,00	14,00	0,00	3,00	5,75
IDCC Desvantagem	2,35	2,92	0,00	12,00	0,00	1,00	4,00
IDCC Defeito	4,25	4,18	0,00	16,00	0,25	3,00	6,00
IDCC Total	10,04	9,19	0,00	41,00	3,25	8,00	12,00
IFV Fadiga e limitação vocal	2,52	2,75	0,00	12,00	0,00	2,00	4,00
IFV Restrição vocal	3,42	2,94	0,00	10,00	1,00	3,00	6,00
IFV Desconforto físico associado à voz	0,63	1,07	0,00	4,00	0,00	0,00	1,00
IFV Recuperação com repouso vocal	7,75	4,21	0,00	12,00	4,00	9,00	12,00
IFV Total	10,83	5,85	0,00	25,00	8,00	11,00	15,00

Análise descritiva

**Legenda:** n = frequência absoluta; % = frequência relativa; DP = desvio padrão; 1Q = primeiro quartil; 3Q = terceiro quartil; IDV-10 = Índice de Desvantagem Vocal 10; IDCC = Índice de Desvantagem para o Canto Clássico; IFV = Índice de Fadiga Vocal

Observa-se na Tabela 3 que os cantores eruditos que, em suas aulas, dão exemplos aos alunos com a própria voz apresentam maiores escores nos instrumentos IDV-10 (p=0,013), IFV restrição vocal (p=0,011) e IFV escore total (p=0,015).

**Tabela 3:** Análise inferencial de comparação das variáveis autoavaliação vocal, IDV-10, IDCC e IFV em função da variável “em suas aulas, você dá exemplo aos alunos com sua própria voz” em cantores eruditos

Variável	Em suas aulas, você dá exemplo aos alunos com sua própria voz?	Média	DP	Mínimo	Máximo	1Q	Mediana	3Q	p-valor
Autoavaliação vocal	Sim	1,08	0,69	0,00	3,00	1,00	1,00	1,00	0,831
	Não dou aula	1,12	0,82	0,00	3,00	0,75	1,00	2,00	
IDV-10 Total	Sim	1,88	1,99	0,00	7,00	0,00	1,00	2,25	0,013*
	Não dou aula	0,81	1,36	0,00	5,00	0,00	0,00	2,00	
IDCC Incapacidade	Sim	3,73	3,01	0,00	11,00	1,75	3,00	6,25	0,368
	Não dou aula	3,15	3,41	0,00	14,00	0,00	3,00	5,25	
IDCC Desvantagem	Sim	2,73	2,79	0,00	10,00	1,00	1,00	4,25	0,066
	Não dou aula	1,96	3,05	0,00	12,00	0,00	0,00	3,25	
IDCC Defeito	Sim	3,85	3,40	0,00	13,00	1,00	3,00	6,00	0,875
	Não dou aula	4,65	4,87	0,00	16,00	0,00	3,00	7,25	
IDCC Total	Sim	10,31	8,03	1,00	31,00	4,00	8,00	12,25	0,463
	Não dou aula	9,77	10,37	0,00	41,00	0,00	8,50	13,25	
IFV Fadiga e limitação vocal	Sim	2,88	3,17	0,00	12,00	0,00	2,00	6,00	0,553
	Não dou aula	2,15	2,27	0,00	7,00	0,00	2,00	3,25	
IFV Restrição vocal	Sim	4,42	2,83	0,00	10,00	1,75	5,00	6,25	0,011*
	Não dou aula	2,42	2,74	0,00	10,00	0,00	1,50	4,00	
IFV Desconforto físico associado à voz	Sim	0,69	1,16	0,00	4,00	0,00	0,00	1,00	0,665
	Não dou aula	0,58	0,99	0,00	3,00	0,00	0,00	1,00	
IFV Recuperação com repouso vocal	Sim	7,31	3,86	0,00	12,00	4,00	8,00	12,00	0,344
	Não dou aula	8,19	4,58	0,00	12,00	3,75	11,00	12,00	
IFV Total	Sim	12,69	6,29	0,00	25,00	9,50	12,50	16,25	0,015*
	Não dou aula	8,96	4,79	0,00	20,00	5,00	9,00	12,00	

* Valores significativos (p≤0,05) - Teste de Mann-Whitney

**Legenda:** DP = desvio padrão; 1Q = primeiro quartil; 3Q = terceiro quartil; IDV-10 = Índice de Desvantagem Vocal 10; IDCC = Índice de Desvantagem para o Canto Clássico; IFV = Índice de Fadiga Vocal

A Tabela 4 mostra que cantores eruditos que realizam desaquecimento vocal depois de cantar, ministrar aulas de canto ou ensaiar canto apresentam maiores escores no IFV restrição vocal do que os que não o fazem (p=0,016).

**Tabela 4:** Análise inferencial de comparação das variáveis autoavaliação vocal, IDV-10, IDCC e IFV em função da variável realiza desaquecimento vocal depois de cantar, ministrar aulas de canto ou ensaiar canto em cantores eruditos

Variável	Realiza desaquecimento vocal?	Média	DP	Mínimo	Máximo	1Q	Mediana	3Q	p-valor
Autoavaliação vocal	Não	1,09	0,75	0,00	3,00	1,00	1,00	2,00	0,983
	Sim	1,11	0,76	0,00	3,00	1,00	1,00	1,25	
IDV-10 Total	Não	1,35	2,00	0,00	7,00	0,00	0,00	2,00	0,322
	Sim	1,33	1,28	0,00	5,00	0,00	1,00	2,00	
IDCC Incapacidade	Não	3,26	3,23	0,00	14,00	0,00	3,00	5,00	0,559
	Sim	3,78	3,21	0,00	11,00	1,75	3,00	6,25	
IDCC Desvantagem	Não	2,15	2,95	0,00	12,00	0,00	1,00	4,00	0,276
	Sim	2,72	2,93	0,00	10,00	0,75	1,50	4,25	
IDCC Defeito	Não	4,44	4,54	0,00	16,00	0,00	4,00	6,25	0,808
	Sim	3,89	3,50	0,00	13,00	2,00	3,00	5,50	
IDCC Total	Não	9,85	9,61	0,00	41,00	2,50	8,00	12,75	0,664
	Sim	10,39	8,58	2,00	31,00	4,00	7,50	12,25	
IFV Fadiga e limitação vocal	Não	2,38	2,66	0,00	12,00	0,00	2,00	3,00	0,729
	Sim	2,78	2,98	0,00	8,00	0,00	1,50	6,00	
IFV Restrição vocal	Não	2,71	2,68	0,00	10,00	0,00	2,00	5,00	0,016*
	Sim	4,78	3,00	0,00	10,00	1,75	5,50	7,00	
IFV Desconforto físico associado à voz	Não	0,56	0,99	0,00	4,00	0,00	0,00	1,00	0,562
	Sim	0,78	1,22	0,00	4,00	0,00	0,00	1,25	
IFV Recuperação com repouso vocal	Não	7,12	4,37	0,00	12,00	3,75	8,50	12,00	0,122
	Sim	8,94	3,72	1,00	12,00	6,75	10,50	12,00	
IFV Total	Não	10,53	5,32	0,00	25,00	7,75	11,00	14,25	0,623
	Sim	11,39	6,87	0,00	23,00	7,25	10,50	17,00	

* Valores significativos (p≤0,05) - Teste de Mann-Whitney

**Legenda:** DP=desvio padrão; 1Q=primeiro quartil; 3Q=terceiro quartil; IDV-10 = Índice de Desvantagem Vocal 10; IDCC = Índice de Desvantagem para o Canto Clássico; IFV = Índice de Fadiga Vocal

A Tabela 5 compara as diversas correlações significantes de forças variadas entre os protocolos, sendo as mais fortes entre o escore total e o escore incapacidade do IDCC (r = 0,868; p = 0,000) e entre o escore total e o escore defeito do IDCC (r = 0,921; p = 0,000*). Quando se analisa as correlações entre os escores de diferentes protocolos, as correlações significantes mais fortes ocorreram entre IFV fadiga e limitação vocal e escore total do IDV-10 (r = 0,415; p = 0,002*) e entre IFV fadiga e limitação vocal e escore total do IDCC (r = 0,410; p = 0,003*), o que mostra uma possível relação entre autopercepção de fadiga e limitação vocal com o autorrelato de desvantagem vocal geral e específica no canto.

## DISCUSSÃO

O canto erudito é um estilo dentro da arte do canto que requer absoluto domínio emocional e um preciso controle de todo o aparelho fonatório^(11)^. A harmonia entre essas consciências proporciona não apenas uma rica interpretação vocal, a que denominamos técnica, mas também enchem a audiência e o próprio intérprete de prazer e, consequentemente, qualidade de vida^(23)^.

Cantores são descritos como a elite dos profissionais da voz, tal qual atores e professores, sendo um corpo social cujo principal instrumento de trabalho é a voz, o que torna o tema fadiga vocal comum a eles^(18)^. Estudos nos apontam que qualquer mínima alteração percebida por esta comunidade pode causar grande impacto na qualidade de vida^(19)^.

O presente estudo procurou identificar qual a percepção os cantores líricos dos coros profissionais do Brasil têm sobre suas vozes, sua percepção sobre fadiga vocal e a percepção de recuperação, mediante repouso vocal, por meio de instrumentos psicométricos reconhecidos internacionalmente^(24)^ e validados e/ou com equivalência cultural no Brasil^(16,19,21)^.

A equivalência cultural já foi demonstrada ser eficiente para pesquisa em instrumentos traduzidos para outras línguas^(26-29)^, proporcionando aos resultados confiabilidade, possibilidade de reprodução e sensibilidade estatística. Isso os torna significativos para a realidade da demanda vocal que esses profissionais vivenciam no dia a dia^(24)^.

Sendo assim, a importância de um indivíduo se autoavaliar^(20)^ contribui para achados clínicos robustos, além da efetividade na escolha da uma melhor conduta terapêutica^(21,22)^.

Na Tabela 1 deste estudo, podemos inferir que os indivíduos pesquisados possuem amadurecimento técnico-vocal. Esses não relatam se perceberem em desvantagem no canto^(19)^, seja pelos anos de estudo do canto (76,92%) ou pela experiência adquirida através do tempo de atuação e demanda (94,23%)^(19)^. Assim, correspondem também à literatura, ao considerarem ter entre uma boa e ótima voz^(21)^.

A literatura também nos mostra que estes profissionais, com estudo técnico de anos e experiência cotidiana, têm maior percepção sobre si mesmos^(15)^. Embora não seja o objetivo do estudo falar sobre alguma predominância relacionada a gênero e/ou registro vocal, o questionário de caracterização (Tabela 1) ressalta maior participação dos naipes de soprano (n = 13 - 25%), barítonos (n = 12 - 23,08%) e tenores (n = 9 - 17,31%).

A literatura descreve que os ajustes vocais na voz de um cantor lírico são executados por um complexo mecanismo que envolve não apenas a adaptação do trato vocal, preparando-o para a emissão de uma nota musical e pertinente a cada registro^(13)^, mas também a capacidade pneumofonoarticulatória, a qual ajusta a pressão subglótica e o controle da passagem da coluna de ar pela tensão gerada no tórax, dotando a voz do intérprete com riqueza de harmônicos e projeção. Do ponto de vista da articulação, podemos dizer que esta é exigida do cantor pois há a necessidade de ressaltar a inteligibilidade do texto cantado, quase sempre em outro idioma que não do domínio linguístico e fluente do indivíduo. Em muitos casos há a necessidade de sobrearticulação, a depender da altura da notação musical no qual este texto está em função. Lábios, posição de língua, movimentação/abertura de mandíbula, não podem ser restritos e constituem um ajuste articulatório para a eficiência e inteligibilidade de determinados fonemas em outro idioma além do refinamento do canto. Há, portanto, um conjunto de potências (pulmonar, fonatória e articulatória) que necessitam trabalhar em conjunto e harmonicamente. Qualquer mínima imprecisão/incoordenação na execução destes ajustes pode levar o profissional a uma percepção de comprometimento de sua *performance* ^(1,2)^.

No presente estudo há uma correlação de resultados entre alguns dos instrumentos utilizados que nos indicam a percepção de fadiga destes indivíduos. A Tabela 2 apresenta em seus resultados que os indivíduos deste estudo não se percebem em desvantagem vocal (IDV-10: 1,35; IDCC: 2,35)^(4)^. Porém, há correlação àqueles que se referiram perceber incapacidade (3,44) e/ou defeito (4,25) no instrumento IDCC (escore total IDCC neste estudo: 10,04)^(30)^ e IFV Fadiga e limitação vocal: 2,52 (pontuação máxima para este item 4,5) e IFV Restrição vocal: 3,42; pontuação máxima para esse item 3,5)^(16)^.

A Tabela 3 apresenta os resultados para indivíduos que, além da rotina diária, são professores de canto e em aulas dão exemplos aos alunos com a voz. Os valores de p mostram uma proximidade de valores entre os itens que indicam restrição (IDV-10: 1,88; p = 0,06; IFV Restrição vocal: 4,42; p = 0,011). O escore total do IFV para os indivíduos que são professores e dão exemplos aos alunos é de 12,69 (p = 0,015), portanto, acima do escore total para o protocolo, o qual foi estabelecido com nota de corte para este instrumento de 11,5^(16)^.

Também são relevantes neste estudo os achados sobre a percepção de recuperação com repouso vocal (IFV: 10,69; p = 0,015). 53,85% destes indivíduos relatam terem procurado ajuda de fonoaudiólogo devido algum problema de voz e 75% já procuraram tratamento com otorrinolaringologista^(15)^.

A fadiga vocal pode estar associada a uma hiperfunção vocal. É um sintoma relatado quase sempre como esforço vocal e desconforto, faixa de *pitch* reduzida e flexibilidade, projeção ou potência vocal reduzida, controle reduzido da qualidade da voz, aumento dos sintomas na fala ao longo do dia e melhora após repouso. Os resultados obtidos na Tabela 4 mostram que 54% dos cantores que realizam desaquecimento vocal após cantar e/ou ministrar aulas de canto nas quais dão exemplos aos alunos com a própria voz apresentam maiores escores nos instrumentos IFV restrição (p = 0,016)^(16,17)^. Embora o desaquecimento vocal seja descrito na literatura como o ajuste que proporciona à musculatura o retorno ao padrão vocal coloquial, não há consenso sobre seus efeitos em outras práticas como a medicina do esporte, fisioterapia e educação física. Acredita-se que o desaquecimento vocal promova a dissipação de resíduos musculares, sobretudo o ácido lático, com redução da dor muscular pós exercício de 24 a 48 horas. Sendo assim, sob o ponto de vista de autopercepção dos indivíduos deste estudo, o desaquecimento vocal mesmo sendo realizado após a atividade do canto/aulas de canto, não lhes propiciou efetiva e imediata sensação de conforto à voz que outrora encontrava-se numa condição de hiperfunção.

Como limitação, o presente estudo foi coletado no período da pandemia do COVID-19 com os cantores em “*lockdown”,* portanto, não vivenciando diariamente a prática de ensaios e/ou sua rotina de ministrar aulas presenciais, mantendo as suas atividades laborais virtualmente.

## CONCLUSÃO

Cantores eruditos profissionais neste estudo não apresentam desvantagem vocal no canto. Porém, questões relacionadas à autopercepção de fadiga vocal se mostram mais presentes quando associadas às atividades além do uso da voz cantada, como dar exemplos em aulas com a voz. Ter tido problema vocal e procurado fonoaudiólogo no passado proporciona maior percepção de recuperação vocal com repouso.
